# Cochlear Implant Reliability: Reporting of Device Failures

**DOI:** 10.1007/s12070-020-01826-9

**Published:** 2020-03-07

**Authors:** Graham O’Neill, Neil S. Tolley

**Affiliations:** Dept of Otolaryngology, Head and Neck Surgery, St Mary’s Hospital, London, UK

**Keywords:** Implanted device, Reliability, Cumulative failure percentage, Cumulative failure rate, Instantaneous failure rate

## Abstract

The aim of this study was to investigate the adequacy of the reporting of cochlear implant device failures. Data from a parallel study involving over 6300 children [1] was used to calculate the instantaneous failure rate for explantations. We found that this is comparable to what manufacturers term ‘Cumulative Failure Percentage’ (CFP). This finding raises concerns about the information provided by manufacturers on the reliability of their implants.

## Introduction

As indicated in a parallel study [1], cochlear implant device failures as reported in clinical studies are generally much higher compared to figures quoted by manufacturers. In particular Wang et al. [2] (e.g. their Fig. 1b), show very significant differences (a multiple of about × 2.5) compared to the manufacturer’s data for the equivalent patient group. Similar results are evident from a study of device failure rates in a paediatric population [3] but here, the ‘overall’ clinical results require transformation to ‘patient time’ for the appropriate comparison to be made. Interestingly even one Cochlear Implant manufacturer, referring to reporting methods of competitors, mentions some very large differences (up to a multiple of × 5) between clinical results and manufacturers’ reported figures [4]. Technical evaluation of explanted devices is the responsibility of the manufacturer which clearly has the potential of selective reporting. Also, whilst ‘device failure’ is differentiated from ‘accidental damage’ it may be argued that the latter could still be a device-related fault vis-à-vis low impact resistance. Furthermore, as one manufacturer states, there are considerable differences in reliability figures depending upon whether or not accidental damage and medical/surgical reasons for explantation (‘extended CSR data’) are included [5]. Unfortunately, and as indicated by a competitor [6], the manufacturer only publishes ‘combined’ data i.e. no separate data for children and adults. This is an important consideration given that device failure in the paediatric population is significantly higher (≃ × 2) than that reported for adults [2, 3]. Considering the above, there is considerable scope for what is, and how it is, reported. We took a closer look at these issues as described below.

## Methods

We decided to compare the instantaneous failure rate for explantations from our parallel study [1] with one manufacturer’s implant data which it refers to as ‘Cumulative Failure Percentage’ (CFP). We also decided to check ‘Cumulative Survival Rate’ calculations from a clinical study which encourages use of this metric.

### Ethical Considerations

This is a “service review” of published data relating to CI reliability. It does not involve patient study or contact. Ethical review was not required.

## Results

**Fig. 1:**
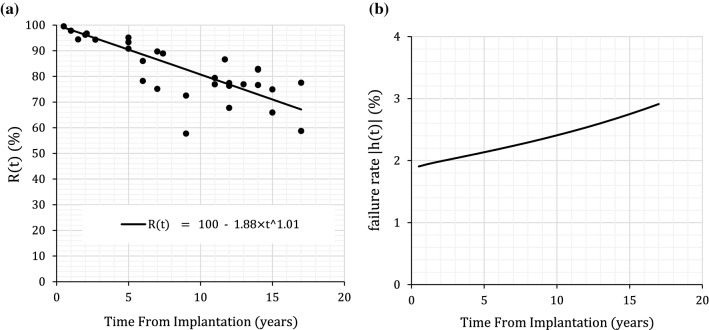
**a** Reliability function for Clinical Data—Explantations. Let unreliability function F(t) = cumulative explantations = EXPc(t) (ref 1). Thus, Reliability R(t) = 100–F(t) = 100–1.88*t*^1.01^. **b** Instantaneous failure rate for Clinical Data—Explantations. $h(t) = \frac{dR(t)/dt}{{R(t)}} = \frac{{d(100 - 1.88t^{1.01} )/dt}}{R(t)} = \frac{{ - 1.9t^{0.01} }}{{100 - 1.88t^{1.01} }}$ A progressive increase in unreliability (increasingfailure rate with respect to the length of time following implantation) is clearly evident

**Fig. 2:**
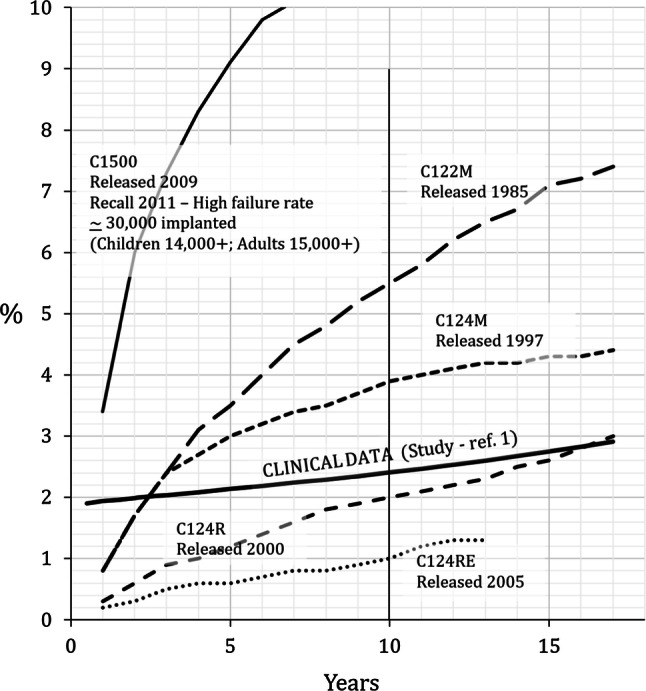
Manufacturers’ ‘Cumulative Failure Percentage’ (CFP) compared to instantaneous failure rate from Clinical Data (ref. 1). The fact that the manufacturers’ data ‘straddle’ the study results seems to suggest that the manufacturers’ data are yearly failure rates not ‘cumulative’ values as they claim. Manufacturers’ Data is from Cochlear® Nucleus Implant Reliability Report, Vol 16, Dec 2017 where Cumulative Failure Percentage (CFP) = 100—Cumulative Survival Percentage (CSP) (see Cochlear® Reliability Report, Vol12, Feb 2014 (p3) for confirmation of this formula)

Figure 1b shows the instantaneous failure rate for explantations calculated from the explantation reliability data shown in Fig. 1a. A marked increase in the failure rate with length of use post-implantation is clearly evident. Figure 2, remarkably, shows a considerable degree of similarity between the instantaneous failure rate for explantations with one manufacturer’s implant data which it refers to as ‘Cumulative Failure Percentage’. This would still be the case if allowance was made, on the basis of the clinical evidence, of reducing explantations associated with device failure from 100 to 80%. Thus, it would appear that either the manufacturers’ figures are interval (not cumulative) values or that selective reporting of what the manufacturer considers a ‘device failure’ is of such a magnitude that the cumulative figures are little different to yearly failure rates for explantations.

Regarding the Cumulative Survival Rate (CSR) metric, a clinical study involving implantation over 11 years quotes a cumulative survival estimate of 91.7% (cumulative failure rate = 100 − 91.7 = 8.3%) which exactly matches the overall failure rate of 8.3% [7]. Similarly, for the paediatric group (their Fig. 2), over 9 years the CSR was 85.3%, matched exactly by the overall failure of 14.7%. Logically, the cumulative failure should be greater, possibly much greater, than the overall failure since the latter does not take account of the gradual recruitment of patients throughout the duration of the study. From the information provided in their paper we were able to model their yearly CSR percentages exactly only if the staggered-entry nature of the study was ignored. If accounted for, a simple estimate of the true cumulative failure rate, e.g. by assuming the same number of patients were recruited each year, would give a figure of about 30%. Using the roughly linear increase in patient recruitment which actually took place gives the more accurate figure of 36%.

## Discussion

Whatever the reason it is clear that little, if any, reliance can be placed upon manufacturers reliability reports. In these reports reference is made to international standards (e.g. ISO 5841–2) [8] but unfortunately there is a deficiency of technical clarity, particularly of distinguishing between an ‘interval’ (conditional) probability (*p*_*i*_) and the ‘cumulative’ probability which is the product of all preceding interval probabilities (= $\prod_{j=0}^{i}$*p*_*j*_). This is a surprising omission, not only because of the technical nature of the data which is being presented but of at least one reliability report where the ‘Cumulative Survival Rate’ (CSR) is explained in terms which can be interpreted as an interval probability [9]. The clinical example provided above of the CSR (= CSP) data raises concerns regarding the extent of erroneous reporting in the relevant literature using this metric.
